# Distribution of salicifoline in freeze-fixed stems of *Magnolia kobus* as observed by cryo-TOF-SIMS

**DOI:** 10.1038/s41598-017-06444-0

**Published:** 2017-07-19

**Authors:** Wakaba Okumura, Dan Aoki, Yasuyuki Matsushita, Masato Yoshida, Kazuhiko Fukushima

**Affiliations:** Graduate School of Bioagricultural Sciences, Nagoya University, Furo-cho, Chikusa-ku, Nagoya, Aichi 464-8601 Japan

## Abstract

Alkaloids are basic nitrogen-containing chemicals that have important physiological and pharmacological characteristics. Many vascular plant species contain alkaloids, and their roles *in planta* are of interest. However, the detailed distribution of alkaloids remains unclear because of their low water solubility and low concentrations in plants. In this study, we visualized the distribution of salicifoline, a water-soluble quaternary ammonium alkaloid, in the freeze-fixed stems of *Magnolia kobus* by cryo time-of-flight secondary ion mass spectrometry. Most of the salicifoline was distributed in living phloem tissues. In the xylem, salicifoline was detected in ray cells, lignifying wood fibres, and in vessels in the latest annual ring. The salicifoline distribution in the xylem varied with the cell wall formation stage. These results provide new insights into the storage, transportation, and role of the alkaloid salicifoline in *M. kobus*.

## Introduction

Alkaloids are characteristic chemicals with interesting physiological and pharmacological properties, and they play an important role in the biological functions of plants. For example, in the old plant order Magnoliales, several alkaloids with antibacterial and insecticidal activities have been reported^1–5^. The active characteristics of alkaloids are also valuable for humans, and their quantities, chemical structures, and pharmacological characteristics have long been studied^1, 4, 6–9^. The significant characteristics of alkaloids have been studied using extracted and purified materials; however, their distribution and roles *in planta* are not completely understood. This poor understanding is partly because of the low concentration of alkaloids in plants and the low water solubility of alkaloids. The poor water solubility results in the loss of positional information during sample preparation processes for microscopic observations, such as trimming, histological fixation, sectioning, embedding, dyeing, and drying.

For the *in planta* visualization of these water-soluble chemicals at a microscopic resolution, one of the simplest and most promising approaches is to freeze-fix plant samples and analyse them with imaging mass spectrometry^10–19^. The authors have developed an analytical system that consists of a cryo time-of-flight secondary ion mass spectrometer (cryo-TOF-SIMS), a cryo electron scanning microscope (cryo-SEM), a cryo glove box containing a sliding microtome for the pretreatment of sample surfaces, and a cryo-vacuum shuttle for the transfer of samples^18, 20^. TOF-SIMS is a static and non-destructive surface analysis method that measures only a few nanometres depth from the surface^21^. Further study with the same sample can subsequently be conducted using other techniques.

*Magnolia kobus* DC. is a member of Magnoliales, and the relatively simple tissue structure of this order has been studied^22–24^. *M. kobus* has been reported to contain the quaternary ammonium alkaloid salicifoline^25^. However, the detailed *in planta* distribution of this alkaloid is unknown. In the present study, the distribution of salicifoline in freeze-fixed stems of *M. kobus* was successfully visualized by cryo-TOF-SIMS/SEM analysis at microscopic resolution. The amount and rough distribution of salicifoline were confirmed via quantitative chromatography measurements. To interpret the distribution of salicifoline in the context of the plant physiology, other bioactive substances of potassium and phosphatidylcholine were also visualized. Furthermore, we discussed the salicifoline distribution in context of the detailed classification of phloem and xylem tissues. We also determined the cell wall formation stages using microscopic observations via visible, polarized, and UV light.

As revealed by our study, in the freeze-fixed stem of *M. kobus*, salicifoline was distributed in the cortex, secondary phloem and ray cells in the phloem region. Salicifoline was also observed in the ray cells of the xylem region. Furthermore, salicifoline was detected in the lignifying wood fibres and vessels in the latest annual ring. The salicifoline distribution in wood fibres and vessels varied with the cell wall formation stage. These results offer new insights for the *in planta* distribution of the alkaloid salicifoline in *M. kobus*.

## Results

### Cryo-TOF-SIMS standard spectra

To determine the characteristic secondary ion of salicifoline (C_12_H_20_NO_2_; exact mass: 210.149), standard chemicals were measured by cryo-TOF-SIMS. Different secondary ions are produced under different chemical conditions during TOF-SIMS measurements because of the “matrix effect”^21^. Potassium is the most abundant inorganic metal in plants^26, 27^ and can readily ionize and affect the coexisting organic chemicals in TOF-SIMS measurements. Therefore, KCl was added to aqueous solutions of the standard chemicals to imitate the chemical environment in the plants.

**Figure 1:**
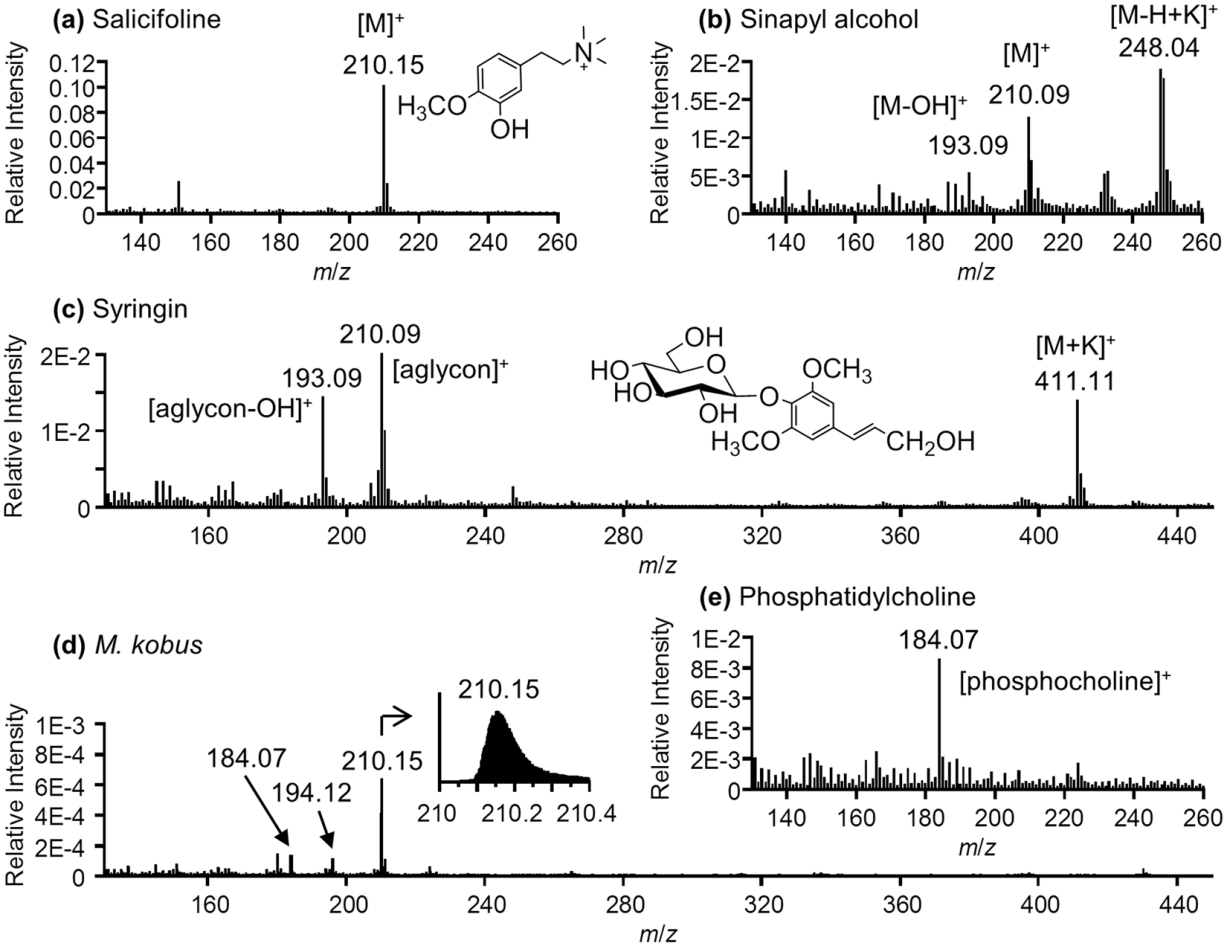
Cryo-TOF-SIMS spectra of (**a**) salicifoline, (**b**) sinapyl alcohol, (**c**) syringin, and (**e**) phosphatidylcholine. A typical cryo-TOF-SIMS spectrum obtained from the transverse surface of the freeze-fixed stem of *M. kobus* is shown in (**d**). Standard chemicals were dissolved at 100 mM concentration in 100 mM aqueous solution of KCl and frozen for the measurements. All spectra were obtained in bunched mode and are displayed as relative intensity to total ion counts.

In this study, sinapyl alcohol (C_11_H_14_O_4_; exact mass: 210.089) and syringin (C_9_H_24_O_9_; exact mass: 372.142) were measured as standard chemicals whose secondary ion peaks had possible signal overlap with salicifoline. The resultant standard cryo-TOF-SIMS spectra are shown in Fig. 1. A typical cryo-TOF-SIMS spectrum obtained from the transverse surface of the freeze-fixed stem of *M. kobus* is also displayed in Fig. 1d.

The strongest secondary ion of salicifoline had a *m*/*z* of 210.15 ion and represented a molecular ion [M]^+^ (Fig. 1a). In the spectrum of sinapyl alcohol (Fig. 1b), secondary ions with a *m*/*z* of 248.04 for [M–H + K]^+^, *m*/*z* of 210.09 for [M]^+^, and *m*/*z* of 193.09 for [M-OH]^+^ were detected, and the ion with a *m*/*z* of 248.04 ion was strongest. Syringin produced secondary ions with *m*/*z* of 411.11 for [M + K]^+^, *m*/*z* of 210.09 for its aglycon unit, and *m*/*z* of 193.09 for the corresponding [aglycon-OH]^+^ (Fig. 1c), and their detection intensities were similar. These results show that sinapyl alcohol and syringin were readily distinguished from salicifoline by their potassium adduct ions at *m*/*z* 248.04 and 411.11, respectively.

As shown in Fig. 1d, the actual and typical cryo-TOF-SIMS spectrum obtained from the transverse surface of the freeze-fixed stems of *M. kobus* exhibited a strong *m*/*z* 210.15 ion and no significant ions with *m*/*z* 411, 248, and 193, and this finding was observed for all measured regions. This result suggests that the *m*/*z* 210 ion detected at the sample surface was primarily derived from salicifoline. The ionization behaviour of these chemicals will be discussed later along with the actual amounts in the stem of *M. kobus*.

Additionally, an aqueous solution of phosphatidylcholine ([C_5_H_13_NO_4_P][lipids]_2_) was measured by cryo-TOF-SIMS. Phosphatidylcholine is a main component of biological membranes in plant cells^28^, and reports have shown that the *m*/*z* 184.07 ion can be detected as a phosphocholine ([C_5_H_15_NO_4_P]^+^) fragment in TOF-SIMS measurements using dried biomaterials^29–31^. In the cryo-TOF-SIMS spectrum of phosphatidylcholine (Fig. 1e), the *m*/*z* 184.07 ion was detected as a [phosphocholine]^+^ ion. Therefore, *m*/*z* 184.07 ion mapping can be used to visualize the cells with biological membranes. The distribution should represent living cells at the measured surface of the freeze-fixed plant.

### HPLC quantification of salicifoline and syringin

**Figure 2:**
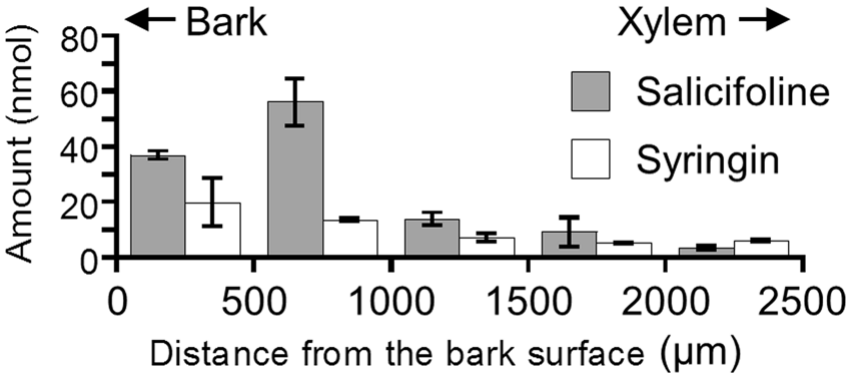
Radial distribution of salicifoline and syringin evaluated by HPLC using serial tangential sections of sample blocks (thickness, 10 mm; circular sector of radius, 5 mm; and central angle, π/16). Measurements were performed in triplicate using three different sample blocks cut from the same disk.

The actual amounts and rough distribution of the target chemicals were quantified using high-performance liquid chromatography (HPLC) measurements of serial tangential sections of freeze-fixed stems of *M. kobus* (Fig. 2). Sinapyl alcohol was barely detected in the sample. Salicifoline and syringin were detected in large amounts in the bark region and in small amounts in the xylem region; their abundance was similar and on the order of several tens of nanomoles per 500 µm thickness.

In the TOF-SIMS measurements, the secondary ion yield depended on the ionization efficiency of the sputtered particle, and the differences could vary over several orders of magnitude^32–34^. In the case of organic compounds, the ionization efficiency might be lower than 1%. Therefore, the positively charged quaternary ammonium alkaloid salicifoline with 100% ionization efficiency showed much higher detectability than the non-ionic syringin. In fact, the cryo-TOF-SIMS spectrum at the transverse surface of the freeze-fixed stem of *M. kobus* (Fig. 1d) showed a strong intensity of the *m*/*z* 210.15 ion and no significant intensities of the *m*/*z* 193.09 and *m*/*z* 411.11 ions over the entire measured regions. From these results, we conclude that the *m*/*z* 210.15 ion could be used to visualize the distribution of salicifoline and the amount of syringin was not sufficient to visualize the distribution in freeze-fixed stems of *M. kobus* in this study.

### Distribution of potassium, phosphatidylcholine, and salicifoline in xylem

**Figure 3:**
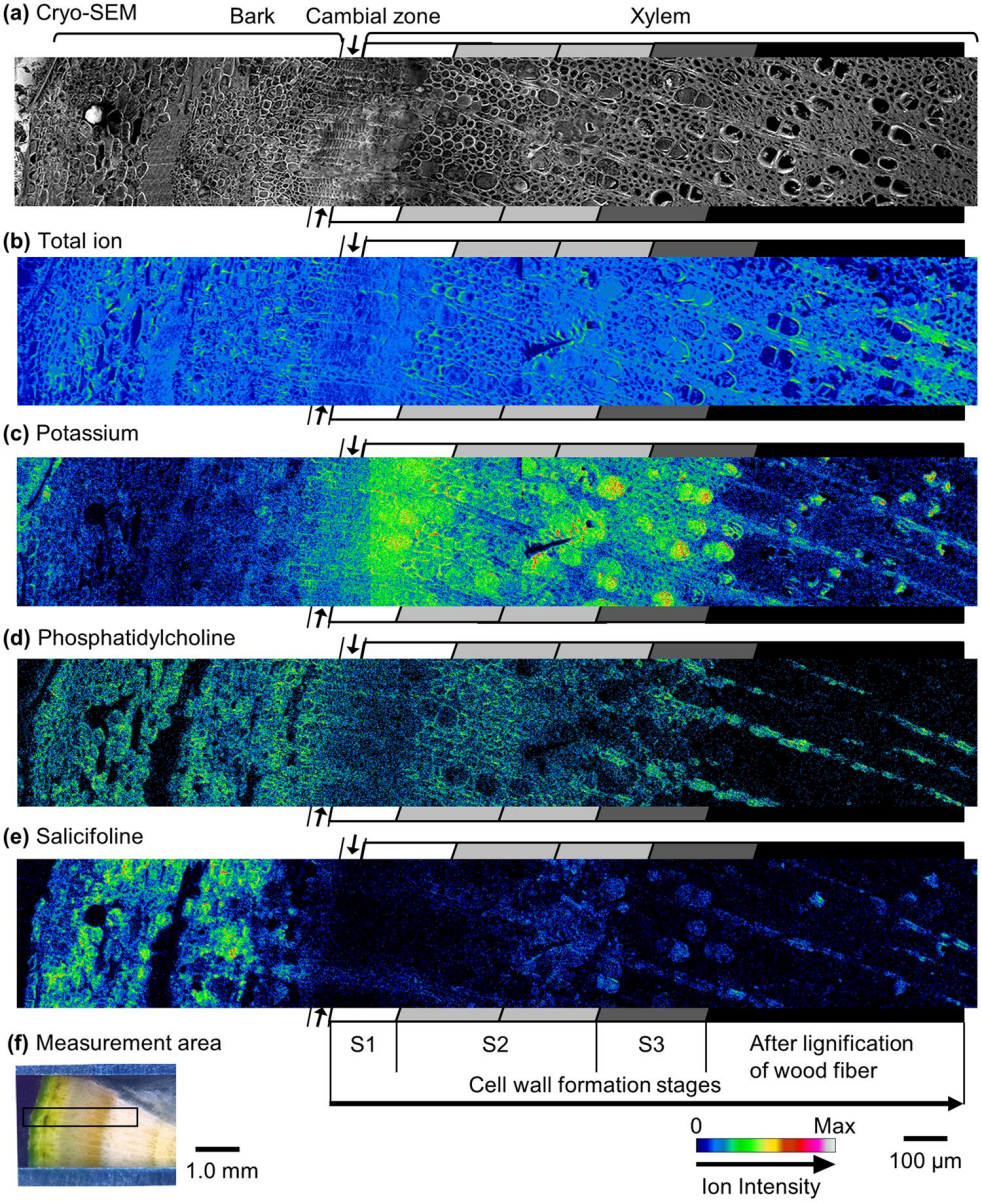
Transverse surface images of freeze-fixed *M. kobus* visualized by cryo-TOF-SIMS/SEM. (**a**) Cryo-SEM image taken after cryo-TOF-SIMS measurement and appropriate freeze etching. Cryo-TOF-SIMS positive ion images of the (**b**) total ion, (**c**) potassium, (**d**) phosphatidylcholine, and (**e**) salicifoline. (**f**) Optical microscopic image of a freeze-fixed stem of *M. kobus* in a sample holder showing the measurement area (approximately 2.4 × 0.4 mm). Scale bars are 100 μm for a, b, c, d, and e and 1.0 mm for f. Arrows above and below the sides of the images of a, b, c, d, and e indicate the cambial zone. Grey scaled tetragons at both sides of the images a, b, c, d, and e indicate the cell wall formation stages of wood fibres.

The results of the cryo-TOF-SIMS/SEM analysis of the freeze-fixed stem of *M. kobus* are shown in Fig. 3. After the cryo-TOF-SIMS measurements, the same region of the sample surface was observed using cryo-SEM (Fig. 3a) after appropriate freeze etching (Supplementary Fig. 1). The measured area contained bark, cambial zone, and xylem (Fig. 3f). The cell wall formation stages of wood fibres in the measured surface were confirmed by microscopic observations using visible, polarized, and UV light (Supplementary Figs 2 and 3), and they are annotated in Fig. 3.

Potassium (*m*/*z* 38.96) was distributed over a wide region at the transverse surface (Fig. 3c), and it was detected especially strongly at the differentiating xylem regions from S1 to S3 formation. After the lignification of wood fibre cells, potassium was mainly detected in the ray cells. The potassium distribution appeared to represent the biological activity of the plant cells. However, potassium was detected in part of the lignified vessels and was weakly detected in the lignified wood fibres. Potassium detection in the vessels suggests the axial translocation of potassium^14^, and reports have indicated that potassium could be stored in dead cells of cedar heartwood^35–39^.

In contrast, phosphatidylcholine, a main component of plant bio membranes^28, 40, 41^, was detected only in living cells (Fig. 3d). The disappearance of phosphatidylcholine from wood fibres correlated with the end of lignification. In vessels, phosphatidylcholine had already been lost in the S2 forming region, although potassium was still detected. Cell death in vessels is known to occur earlier than in wood fibres^42–45^. Based on these points in the cryo-TOF-SIMS analysis, phosphatidylcholine distribution rather than potassium distribution is a better marker of living cells and tissues.

The salicifoline distribution in the xylem region varied with cell wall formation stages (Fig. 3e). In the cambial zone and in the S1 formation stage, salicifoline was detected only in the ray cells. Within the earlier half of S2 formation stages, salicifoline detection in wood fibres was slightly increased. During the latter half of S2 formation, salicifoline was distributed not only in the ray cells but also in the wood fibres and vessels. In the S3 formation stage, salicifoline was detected in the ray cells and vessels. At the end of the lignification of wood fibres, salicifoline was found in the ray cells and certain vessels. Salicifoline and potassium were often detected in parts of the vessels after the end of the lignification of wood fibres. This phenomenon may result from vessel cavitation followed by concentration at the surface of the cell wall. Vessel cavitation was also suggested by the cryo-SEM image (Fig. 3a, Supplementary Fig. 1).

In the ray cells, the area of salicifoline detection in S1 and the earlier half of the S2 formation stages was wider than that in the S3 formation stage and in the ray cells after the lignification of the wood fibres. The differences may be related to the biological activities of the neighbouring wood fibres in the corresponding regions. The resultant salicifoline distribution in the xylem region is summarized in Table 1.

**Table 1:** Distribution of salicifoline in the xylem region of freeze-fixed stems of *M. kobus*.

Cell types	Cell wall formation stages				
	S1	S2 (earlier)	S2 (later)	S3	After lignification
Wood fibre		+	++		
Vessel			++	++	+
Ray	++	++	++	++	++

Symbols: +, detected a little; ++, detected significantly.

### Distribution of salicifoline in the phloem

**Figure 4:**
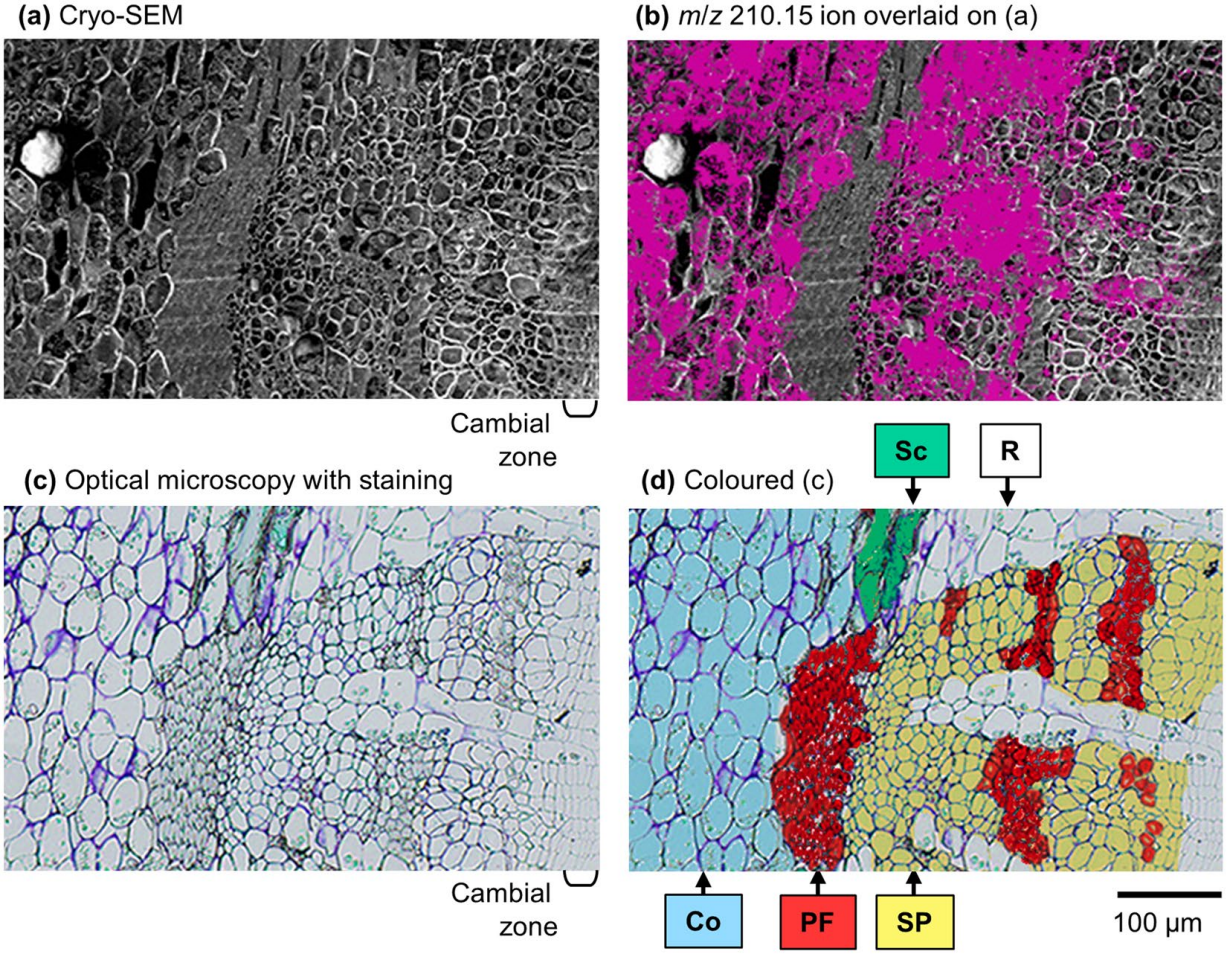
Images obtained by (**a**) cryo-SEM and (**b**) overlay of cryo-TOF-SIMS *m*/*z* 210.15 ion (red) on the cryo-SEM image, illustrating the distribution of salicifoline in the freeze-fixed stem of *M. kobus*. Nearly the same region was observed by (**c**) optical microscopy with toluidine-blue staining, and the assigned tissues were separately coloured to distinguish them (**d**). Letters in d show the positions of cortex (Co), phloem fibre (PF), sclereid (Sc), secondary phloem (SP), and ray cells (R). Scale bar is 100 μm.

As mentioned, most salicifoline was distributed in the phloem region (Figs 2 and 3e). The distribution was further examined with detailed tissue classification using microscopic observations. To clarify the distribution, Fig. 4 displays the overlaid image of the *m*/*z* 210.15 ion onto the cryo-SEM image (Fig. 4b) and the coloured optical microscopic image of phloem tissues (Fig. 4d). The sample surface observed via cryo-TOF-SIMS/SEM analysis (Fig. 4a and b) and optical microscopy (Fig. 4c and d) was nearly at the same position as that obtained from the same sample block, and a similar tissue distribution was observed. The tissues were classified as cortex, phloem fibre, sclereid, secondary phloem, and ray cell.

Phloem fibres and sclereids are lignified and dead tissues, and they do not contain salicifoline. However, cortex and ray cells contain salicifoline. In the secondary phloem, the salicifoline content appeared to increase from the inner side (neighbouring cambial zone) to the outer side. As a result, the major living tissues of phloem, such as the cortex, secondary phloem, and ray cells, contained salicifoline, and the amount in the phloem region was much higher than that in the xylem region.

## Discussion

The TOF-SIMS visualization displays the apparent concentration of the target chemicals on the measured surface, indicating that the obtained distribution does not show biosynthetic activity at the measured surface. The target chemicals should be difficult to visualize if they were transferred via a specific tissue with a concentration below the detection limit and/or were not stored in the tissue.

Most of the salicifoline was detected in the phloem region. Storing alkaloids in the phloem region is a reasonable method of taking advantage of the antibacterial and insecticidal activities of alkaloids. In the phloem region of the stem of *M. kobus*, salicifoline was detected only in the living tissues (Fig. 3e). In the xylem region, ray cells contained and/or transferred salicifoline in all instances. In addition, salicifoline was detected in lignified vessels. These results suggest the possibility of dynamic *in planta* radial/axial translocation of salicifoline via ray cells and vessels.

Salicifoline was not stored in dead tissues, such as phloem fibre and sclereid and xylem lignified wood fibre. The wood fibres synthesized and/or stored salicifoline only at the S2 formation period and then were lost. After cell death, salicifoline was not stored in wood fibres. The reason why *M. kobus* requires salicifoline only for wood fibres at the specific cell wall formation stage is unclear. The appropriateness of the salicifoline distribution should be examined further from the viewpoint of the properties of salicifoline. In conclusion, the visualized salicifoline distribution at microscopic resolution offers new insights for the *in planta* behaviour of this alkaloid and should lead to further discussion of its biosynthesis, storage, transportation, and role in *M. kobus*.

## Methods

### Plant materials

The sample disk (thickness 10 mm) was obtained from the lower part of the flesh branch of *M. kobus* on 19 June 2015 in Nagoya, Japan. The disk was cut into small blocks (circular sector of radius 5 mm and central angle π/8 or π/16) containing bark, cambial zone, and xylem. The blocks were quick-frozen in liquid Freon^®^ 22 (DuPont, USA) at −160 °C and stored at −80 °C.

### Reagents

Salicifoline was extracted and purified from 1.25 kg bark of *M. kobus* according to the previously reported method^25, 46, 47^. The chemical structure and purification of salicifoline was confirmed by ^1^H and ^13^C NMR measurements. Sinapyl alcohol and syringin were synthesized using the method described by Terashima *et al*.^48^ Phosphatidylcholine was purchased and used as received (SIGMA-Aldrich, Product No. P 3556).

### HPLC measurements

The freeze-fixed stem block of *M. kobus* (central angle π/16) was cut into serial tangential sections with 100 μm thickness. Five sections were collected in the same plastic tube, and 1 mL of ultrapure water was added to the tube. The sections were extracted at 100 °C for 30 min and at 25 °C for 2 h. The extracts were analysed by HPLC to quantify the salicifoline, syringin, and sinapyl alcohol contents. The HPLC measurements were performed using a Shimadzu SPD-10A apparatus (Shimadzu, Japan). The measuring conditions for salicifoline quantification were as follows: column, TSK-gel ODS-100S (4.6 mm ID × 250 mm, Tosoh Corp., Japan); flow rate, 1 mL/min; mobile phase, (A) 10 mM ammonium acetate buffer pH 5 and (B) acetonitrile. The linear gradient elution was as follows: 5 min, 10% B; 10 min, linear 10–35% B; 5 min, linear 35–50% B; 5 min, linear 50–10% B; 5 min, 10% B; and 10 min, 30% B. The measuring conditions for syringin and sinapyl alcohol quantification were as follows: column, TSK-gel ODS-100S (4.6 mm ID × 250 mm, Tosoh Corp., Japan); flow rate, 1 mL/min; and mobile phase consisting of (A) ultrapure water and (B) MeOH:acetonitrile = 6:1. The linear gradient elution was as follows: 5 min, 10% B; 10 min, linear 10–20% B; 10 min, linear 20–60% B; 10 min, linear 60–10% B; and 5 min, 10% B. The measurements were performed in triplicate using three blocks from the same disk to evaluate the average amount and standard deviation of the target chemicals.

### Cryo-TOF-SIMS/SEM analysis

The details of the manufactured cryo-TOF-SIMS/SEM system were described previously^18, 20^. The frozen sample block (central angle π/8) was fixed in a Cu sample holder via ice embedding. After cutting to form a clean and flat surface in the glove box under a dry N_2_ atmosphere (<−10 °C), the block was transferred to cryo-TOF-SIMS using a cryo-vacuum shuttle. Positive ion images were obtained using cryo-TOF-SIMS (TRIFT III Spectrometer, ULVAC-PHI Inc.). The measurement conditions were as follows: primary ion, 22 keV Au_1_^+^ at a current 5 nA; measurement time, 5 min; raster size, 400 × 400 µm (256 × 256 pixels) for the freeze-fixed stem of *M. kobus* and 200 × 200 µm for the aqueous solution of standard chemicals; pulse width, 1.8 ns (bunched for spectrum) or 13.0 ns (non-bunched for image); mass range, *m*/*z* 0.5–1850; spot size, 1.0 µm in non-bunched mode; and temperature, −120 to −130 °C. A low-energy pulsed electron ion gun (30.0 eV) was used for surface charge compensation. The aqueous solutions of standard chemicals were frozen and measured by cryo-TOF-SIMS in the same procedure in bunched mode.

In the bunched mode measurements of the freeze-fixed stem of *M. kobus*, the target secondary ions of *m*/*z* 184.07 [phosphocholine]^+^ and *m*/*z* 210.15 [salicifoline]^+^ were unimodal, suggesting that there was no mass interference at the signals. In the case of *m*/*z* 38.96 of [K]^+^, the ion peak shape was bimodal in both measurement modes. The bimodal shape of *m*/*z* 38.96 consisted of major [K]^+^ (*m*/*z* 38.96) and minor C_3_H_3_ (*m*/*z* 39.02) ions, and the [K]^+^ ion could be separated in the non-bunched mode measurements. From these results, the authors concluded that it was possible to visualize the target ions in non-bunched mode in this study.

After the cryo-TOF-SIMS measurements, the plant sample block was transferred to cryo-SEM using a cryo-vacuum shuttle, and the same surface was observed after a freeze-etching treatment to enhance the image contrast of the cryo-SEM images because the sample surface maintained its frozen-hydrated state after the cryo-TOF-SIMS measurements. The observation conditions were as follows: acceleration voltage, 1.5 kV; temperature, −120 °C for observation and −90 °C for freeze etching; and the working distance was 10 mm.

All TOF-SIMS data were obtained as ‘RAW’ data files, and a full mass spectrum was recorded at every 256 × 256 pixel points. The obtained images were connected using WinCadence 5.1.2.8 (ULVAC-PHI Inc.) and MATLAB R2014a (MathWorks, Inc.) with the PLS Toolbox 7.5.2 (Eigenvector Research, Inc.) without any ion count normalisation. After image connection, the colour scale was altered using ImageJ software (The National Institutes of Health, USA). The SEM images and overlay images of the selected ions in the SEM image were prepared using Photoshop CS5 Extended (Adobe Systems, Inc.).

### Microscopic observations

To observe nearly the same surface as that measured by the cryo-TOF-SIMS/SEM, after the cryo-TOF-SIMS/SEM analysis, the same frozen block of *M. kobus* was immersed in glutaraldehyde-acetone solution at −80 °C for 3 days, −30 °C for 1 day, 4 °C for 1 day, and then at room temperature. The sample block was embedded in an epoxy resin and cut into sections at a thickness of 1 or 2 μm using an ultra-microtome (HM350; Microm). The section with a 2 µm thickness was stained by toluidine blue and observed using an optical microscope (BX50; Olympus Corp.). The 1 µm sections were submitted to polarized optical microscopy (BX50; Olympus Corp.) and UV microspectrophotometry (MPM800; Carl Zeiss) observations.

## Electronic supplementary material

Supplementary Information
