# Supplementary material for: Distribution of salicifoline in freeze-fixed stems of Magnolia kobus as observed by cryo-TOF-SIMS
Source: Sci Rep. 2017 Jul 19;7:5939. doi: 10.1038/s41598-017-06444-0 (PMC5517595; doi:10.1038/s41598-017-06444-0)
Supplement: Supplementary file 1 — Supplementary Information [file 41598_2017_6444_MOESM1_ESM.pdf]

Supplemental Information

Distribution of salicifoline in freeze-fixed stems of *Magnolia kobus* as observed by cryo-TOF-SIMS

Wakaba Okumura[1], Dan Aoki[1*], Yasuyuki Matsushita[1], Masato Yoshida[1], and Kazuhiko Fukushima[1]

[1] Graduate School of Bioagricultural Sciences, Nagoya University, Furo-cho, Chikusa-ku, Nagoya, Aichi 464-8601, Japan

[*] Corresponding author
E-mail address: daoki@agr.nagoya-u.ac.jp

Keywords: salicifoline, cryo-TOF-SIMS, *Magnolia kobus* DC., imaging mass spectrometry, alkaloid

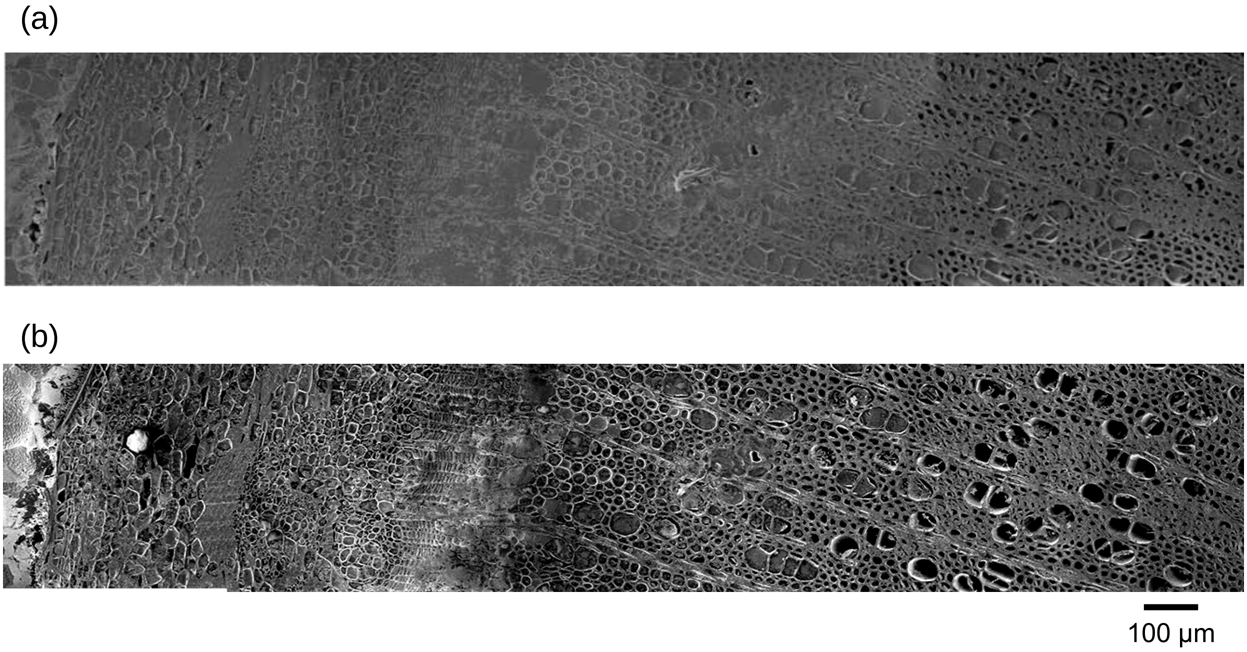

Supplementary Figure 1. Cryo-SEM images (a) just after cryo-TOF-SIMS measurements and (b) after the freeze-etching treatment. Scale bar is 100 μm.

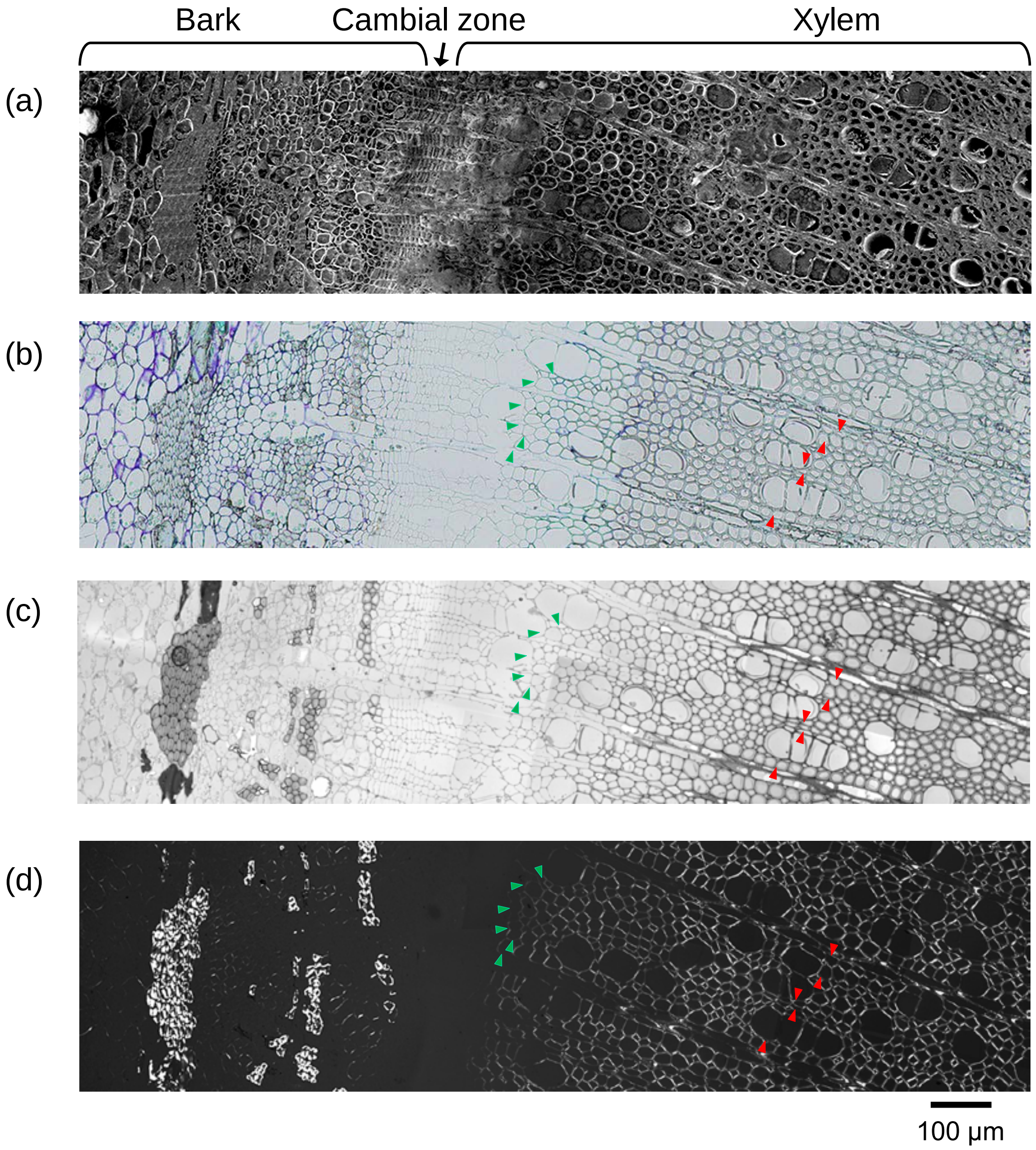

Supplementary Figure 2. Images of transverse section of (a) freeze-fixed stem of *M. kobus* by cryo-SEM, and resin-embedded stem of *M. kobus* observed by (b) visible light with toluidine blue staining, (c) polarized light, and (c) UV light. Green arrow heads suggest the S1 layer birefringence, and red arrow heads indicate S3 layer birefringence.

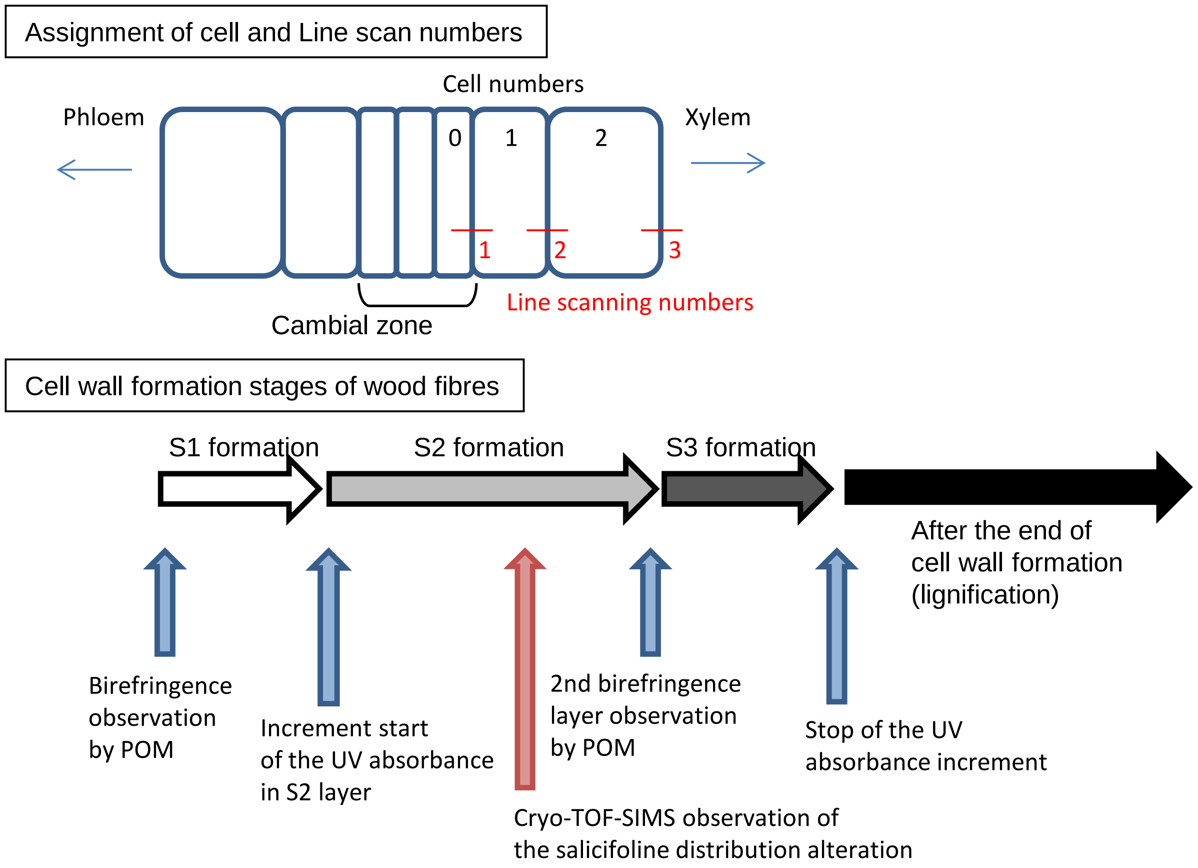

Supplementary Figure 3. Procedure for determination of the cell wall formation stages of wood fibres by polarized optical microscopy (POM) and UV microscopy. The S2 formation stage was divided into two periods with respect to the distribution of salicifoline as visualized through cryo-TOF-SIMS.
